# Supplementary material for: The causal relationship between the human gut microbiota and pyogenic arthritis: a Mendelian randomization study
Source: Front Cell Infect Microbiol. 2024 Nov 26;14:1452480. doi: 10.3389/fcimb.2024.1452480 (PMC11629706; doi:10.3389/fcimb.2024.1452480)
Supplement: Supplementary file 1 [file DataSheet1.zip › mendelian test/ebi-a-GCST90027496.csv_leave_one_out.pdf]

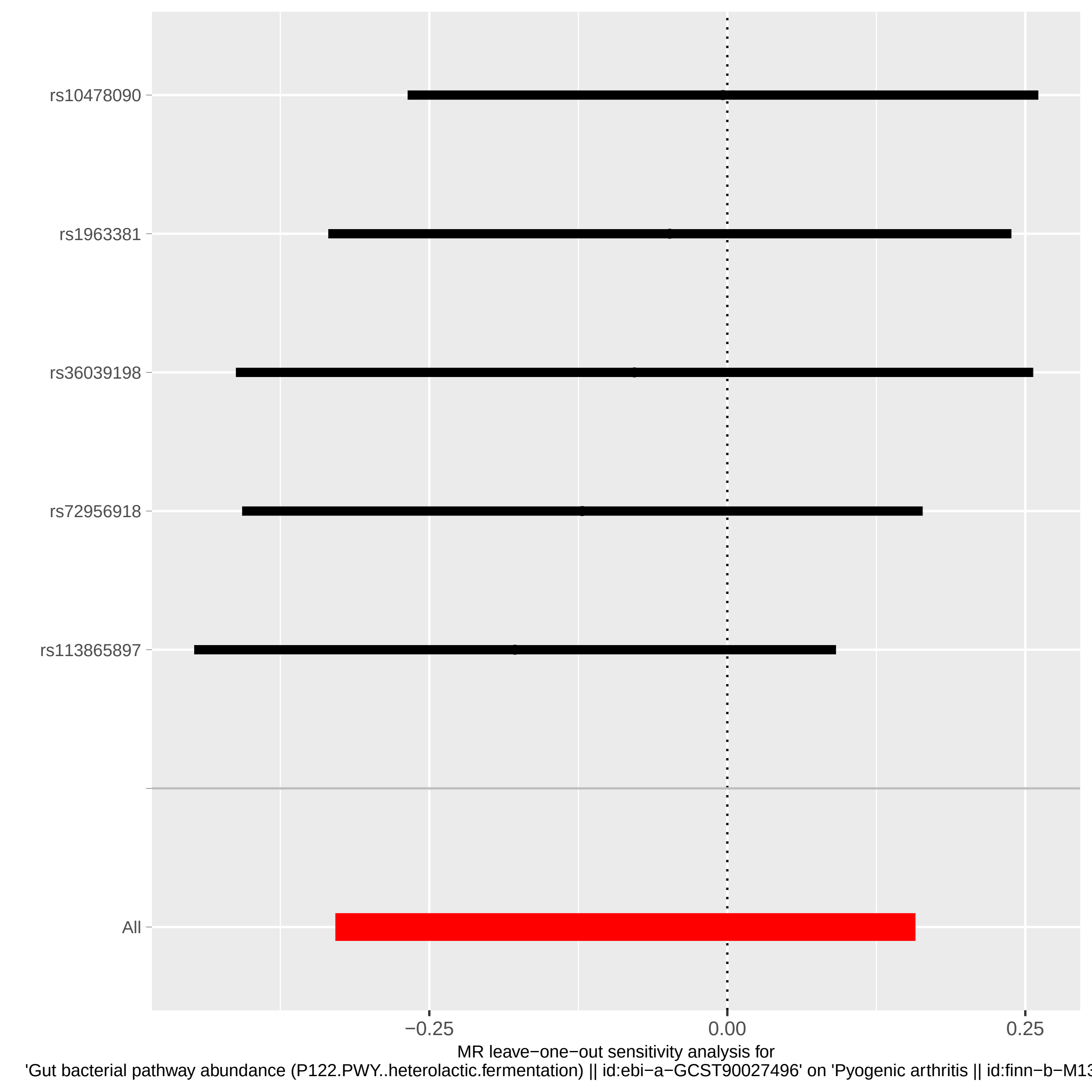
rs10478090
rs1963381
rs36039198
rs72956918
rs113865897
All
−0.25
0.00
0.25
MR leave−one−out sensitivity analysis for
'Gut bacterial pathway abundance (P122.PWY..heterolactic.fermentation) || id:ebi−a−GCST90027496' on 'Pyogenic arthritis || id:finn−b−M13
